# Pediatric Epilepsy Mechanisms: Expanding the Paradigm of Excitation/Inhibition Imbalance

**DOI:** 10.3390/children6020023

**Published:** 2019-02-05

**Authors:** Li-Rong Shao, Christa W. Habela, Carl E. Stafstrom

**Affiliations:** Division of Pediatric Neurology, Johns Hopkins University School of Medicine, Baltimore, MD 21287, USA; lshao5@jhmi.edu (L.-R.S.); chabela1@jhmi.edu (C.W.H.)

**Keywords:** seizures, epilepsy, excitation, inhibition, synaptic function, genetic mutations, *STXBP1*, antiseizure drugs, levetiracetam, metabolism, ketogenic diet

## Abstract

Mechanisms underlying seizures and epilepsy have traditionally been considered to involve abnormalities of ion channels or synaptic function. Those considerations gave rise to the excitation/inhibition (E/I) imbalance theory, whereby increased excitation, decreased inhibition, or both favor a hyperexcitable state and an increased propensity for seizure generation and epileptogenesis. Several recent findings warrant reconsideration and expansion of the E/I hypothesis: novel genetic mutations have been identified that do not overtly affect E/I balance; neurotransmitters may exert paradoxical effects, especially during development; anti-seizure medications do not necessarily work by decreasing excitation or increasing inhibition; and metabolic factors participate in the regulation of neuronal and network excitability. These novel conceptual and experimental advances mandate expansion of the E/I paradigm, with the expectation that new and exciting therapies will emerge from this broadened understanding of how seizures and epilepsy arise and progress.

## 1. Seizure Generation: Hyperexcitability and Hypersynchrony

**Figure 1:**
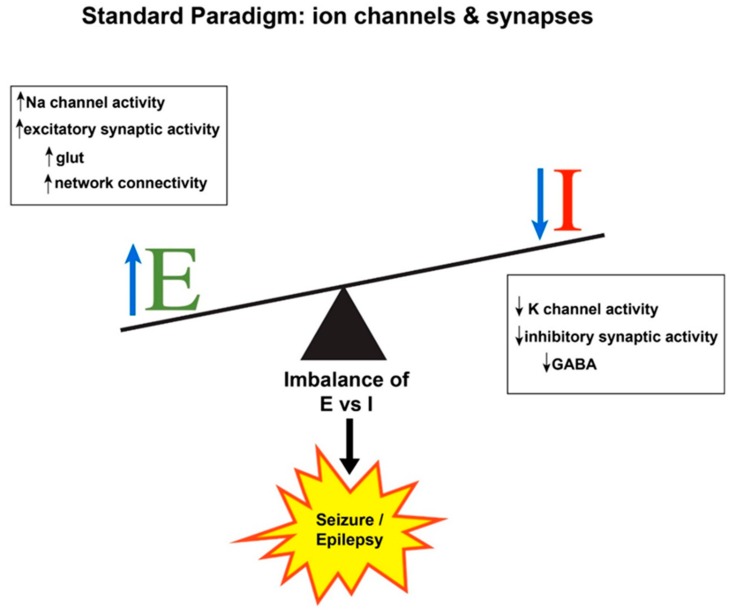
Schematic showing standard paradigm for understanding the balance between excitation (E) and inhibition (I) in the production of seizures and epilepsy. Any physiological change that increases E or decreases I (or both) will tip the balance toward excitation and possible seizure occurrence. GABA, gamma-aminobutyric acid; glut, glutamate; Na, sodium; K, potassium.

The pathophysiological hallmarks of seizure generation are hyperexcitability of individual neurons and hypersynchronous firing of neuronal networks [1]. Hyperexcitability implies that seizure “threshold”, the level of membrane depolarization that must be exceeded for a seizure to occur, is lowered, making it easier for a neuron to fire recurrent discharges that comprise the electrographic manifestations of a seizure. Interconnected networks of hyperexcitable neurons can cause a predisposition to excessive firing as well [2]. Hyperexcitable phenomena include interictal discharges, seizures, and pathological network oscillations [3]. The term “hypersynchronous firing” refers to a population of neurons discharging at the same time [4,5]. While an individual neuron might fire in an epileptic pattern (i.e., rapid, repetitive, paroxysmal discharges), a seizure is inherently a network phenomenon that must entail numerous neurons firing simultaneously. Any brain region can potentially generate a seizure under the appropriate conditions, that is, when net excitation (E) in a cortical area exceeds net inhibition (I) in that area (Figure 1). Each step in the sequence of seizure initiation, propagation, and termination is ultimately governed by the balance between excitation and inhibition, which is best envisioned as a dynamic interaction between neurons, glia, vascular components, and the extracellular milieu.

Historically, seizure generation (ictogenesis) has been considered to represent an imbalance between neuronal excitation and neuronal inhibition [6,7]. This concept, derived from early recordings of synaptic potentials and action potentials [8], has been very useful over the years for understanding the physiological basis of ictogenesis and antiseizure drug (ASD) mechanisms. The excitation/inhibition (E/I) balance would tip toward hyperexcitation if any physiological condition increased glutamatergic synaptic activity (e.g., seizure-induced sprouting or increased connectivity between excitatory pyramidal neurons) or ion currents mediating membrane depolarization (e.g., inward Na^+^ or Ca^2+^ flux). In addition, the balance would be tilted by any situation in which gamma-aminobutyric acid (GABA)-ergic inhibitory synaptic activity is decreased (e.g., seizure-induced loss of inhibitory interneurons) or ion currents mediating membrane hyperpolarization are strongly activated (e.g., outward K^+^ or inward Cl^−^ flux) (Figure 1). The E/I changes may be phasic or tonic, with implications for ictogenesis, epileptogenesis, and their potential treatment [9]. Likewise, most of the currently available antiseizure drugs (ASDs) are aimed at restoring E/I balance by serendipity or design, via opposite actions, i.e., decreasing excitation or increasing inhibition. While this construct has been informative about seizure generation, it has long been recognized as oversimplified [10]. Just as there are numerous seizure types and epilepsy syndromes and etiologies, loss of inhibition is not universal and some types of seizure occur when inhibition is enhanced [5,11,12,13,14]. For example, the generalized spike–wave discharges recorded on an EEG during an absence seizure entail hypersynchronous inhibition in thalamocortical circuits [10,15]. Furthermore, while acute E/I imbalance can be invoked to understand seizure occurrence, it may not be accurate to ascribe seizures in established epilepsy merely to chronic E/I imbalance, as other membrane and circuit homeostatic changes may occur during epileptogenesis [16,17]. Moreover, E/I balance is not static and can change over time [5].

Newer evidence suggests that the E/I imbalance concept requires revision, or at least expansion, for several reasons. There is burgeoning knowledge of genetic mutations responsible for epilepsy, many of which are not directly involved in either synaptic transmission or ion channel function [18] or mediate an excitability change in the opposite direction of what fits in the classic E/I imbalance model. For example, in Dravet syndrome, caused by a loss-of-function mutation of the sodium channel *SCN1A* gene, the primary pathophysiology involves reduced inhibition onto excitatory pyramidal cells (a phenomenon called “disinhibition”) [19]. Likewise, epilepsy is seen in several gain-of-function mutations of potassium channels genes that would be predicted to decrease excitation [20], such as a severe form of childhood focal epilepsy known as malignant migrating partial seizures of infancy in which *KCNT1* gain-of-function mutations are causative [21]. The newly emerging diversity of potential mechanisms encompassed by these genetic mutations greatly expands our understanding of the neurobiological underpinnings of seizures and epilepsy, and also challenges many preconceived notions. In the new era of precision medicine, in which treatment is targeted in patients with specific mutations, epilepsy therapy will also undergo a paradigm shift. In this review, we briefly describe some of the newer concepts related to the pathogenesis of seizures and epilepsy, by providing examples of situations in which the E/I balance does not fully explain the clinical scenario. Our coverage is not comprehensive, of course, and many other examples could have been chosen.

Even the normal brain is poised in a constant state of seizure vulnerability. This notion is supported by the observation that exposures or pathologies that slightly increase excitation or reduce inhibition are ictogenic. Anyone can have a seizure in the right (or wrong!) circumstances, for example, due to ingestion of a toxin, acute metabolic disturbance such as hypoglycemia, or cortical irritability from an infection. An illustrative example is domoic acid ingestion (e.g., from contaminated shellfish), which causes acute seizures in several species, including humans, by over exciting kainic acid receptors, a subtype of glutamate receptor that mediates fast excitatory neurotransmission [22]. Long-term domoic acid exposure may also lead to epileptogenesis and chronic seizures, illustrating the overlap or progression from the initial seizures to circuit damage to epilepsy [23].

When considering chronic epilepsy and its numerous, heterogeneous causes and manifestations, the E/I imbalance theory is especially challenged. It might be reasoned that patients with a permanently altered E/I imbalance would have seizures constantly, rather than intermittently. In addition, as elaborated below, many recently discovered epileptogenic mutations would not lead to the prediction of increased excitation or decreased inhibition, and mutation-specific alterations of excitability can affect different brain areas at different developmental time points [24]. In other disorders in which E/I imbalance has been proposed, such as autism, the E/I hypothesis is also being reconsidered [25]. 

Epilepsy is a disorder of neuronal networks, with the ultimate pathophysiology emerging from patterns of connectivity in different brain regions. The emergent phenomenon of hypersynchrony is also undergoing revision; whereas it was previously envisioned that a focal seizure begins when neurons in a local area fire hypersynchronously, recent research is showing that the situation is more complex. Neuronal firing in a local region may actually be *desynchronized* at seizure onset, and then become more synchronous as the seizure progresses. Further details about this fascinating topic are elaborated in other publications [2,26]. 

## 2. Expanding the E/I Imbalance Paradigm

### 2.1. Newly Discovered Gene Mutations Responsible for Epilepsy Defy Traditional E/I Mechanisms

**Figure 2:**
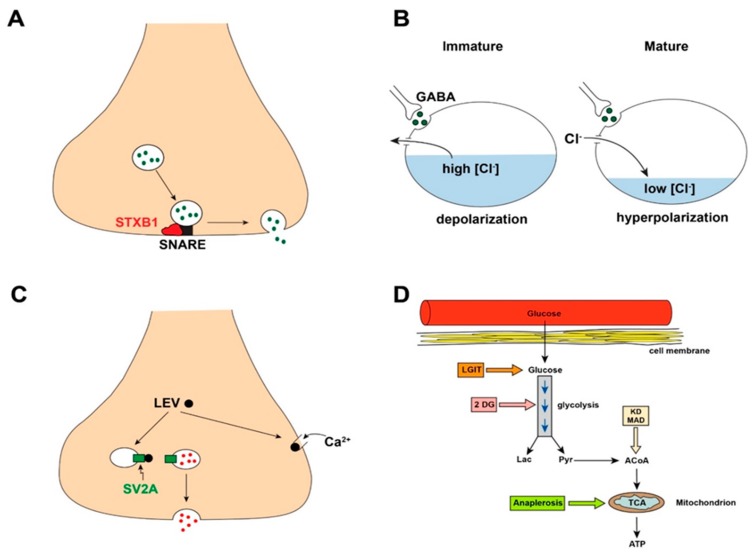
Selected examples of epilepsy mechanisms in which the E/I balance concept may not be immediately applicable. (**A**) STXBP1 is a protein essential for neurotransmitter vesicle docking and fusion to enable subsequent release of neurotransmitter. This protein binds to the soluble N-ethylmaleimide attachment receptor (SNARE) complex (see text) to allow neurotransmitter release. Mutation of the gene that encodes STXBP1 (*STXBP1*) impairs neurotransmitter release (both excitatory and inhibitory neurotransmitters) and leads to a syndrome of neurodevelopmental disorder and severe epilepsy. (**B**) Early in development, GABA is excitatory rather than inhibitory, related in part to age-specific intracellular chloride concentrations. (**C**) The antiseizure drug levetiracetam (LEV) binds to a synaptic vesicle protein called SV2A, leading to reduced vesicle docking and neurotransmitter release. LEV also inhibits presynaptic N-type calcium channels and release of calcium from intracellular stores. (**D**) Overview of glucose (Gluc) metabolism. Glucose enters the cell from the bloodstream and then undergoes glycolysis for the eventual production of ATP. Metabolic control points for potential epilepsy therapy are indicated in the boxes. STXBP1, syntaxin-binding protein 1; LGIT, low glycemic index treatment; 2DG, 2-deoxyglucose; KD, ketogenic diet; MAD, modified Atkins diet; ATP, adenosine triphosphate; TCA, tricarboxylic acid cycle; ACoA, acetyl-co-enzyme A; Lac, lactate; Pyr, pyruvate.

For several epilepsy syndromes of childhood, mutations are being discovered in genes that bear no obvious relationship to excitability mechanisms as traditionally envisioned [27]. A relevant example is *syntaxin-binding protein 1* (*STXBP1),* a gene that regulates syntaxin binding protein 1, which is essential for presynaptic vesicle docking and fusion, necessary steps for neurotransmitter release (Figure 2A) [28,29]. Syntaxin is a component of the soluble N-ethylmaleimide attachment receptor (SNARE) protein complex. Mutation of *STXBP1* causes a severe neurodevelopmental disorder and epilepsy with multiple seizure phenotypes including Ohtahara syndrome and other developmental epileptic encephalopathies. Mutation of *STXBP1* would be predicted to reduce the release of *both* excitatory and inhibitory neurotransmitters, throwing into question why seizures are invariably present in affected children. Recent findings in mice suggest that *STXBP1* mutations might affect inhibitory synapses preferentially, such that the reduction in GABA confers disinhibition and seizures [30,31]. At the same time, *STXBP1*-associated reduction in excitatory transmitter release might be thought to be a seizure-protective mechanism, so the end result of E/I balance in this disorder remains unexplained. The STXBP1 story illustrates how E/I imbalance mechanisms may become more apparent in certain mutations as a field advances.

Numerous other genes similarly affect aspects of synaptic development, neurotransmitter release, subcellular signaling, and other aspects of neuronal function outside the familiar E/I construct. Another example is mutation of the gene for the transcription factor Aristaless-related homeobox protein (ARX), leading to impaired inhibitory interneuron migration from the forebrain ganglionic eminences to the neocortex, resulting in hyperexcitability and propensity to seizures such as infantile spasms [32]. In this case, the E/I balance is upended by diminished inhibitory neuron intercalation into the neocortical circuit. See Table 1 for other selected examples.

### 2.2. Neurotransmitters May Cause Paradoxical Physiological Actions

The recognition that GABA is depolarizing and sometimes excitatory during a window of early brain maturation supports the notion that excitability mechanisms are not as straightforward as previously imagined [33]. This early excitatory GABA effect may seem paradoxical for a neurotransmitter that is generally thought of as inhibitory. However, early GABA-mediated depolarization is critical in the developing brain for trophic functions such as synaptogenesis and circuit development [34]. 

The transition of GABA from excitatory to inhibitory relies on the intracellular chloride concentration (Figure 2B), which is regulated by two chloride-cation membrane co-transporters [35]. The sodium-potassium-chloride co-transporter 1 (NKCC1), maximally expressed early in development (until about term gestational age in humans and 10 days of age in rodents), imports chloride, raising the basal intracellular chloride concentration. Therefore, when GABA binds to its postsynaptic GABA_A_ receptor and opens chloride channels, chloride exits the cell, down its electrochemical gradient. This chloride efflux leads to depolarization of the cell membrane, which can be sufficient to generate action potentials. Over time, NKCC1 expression diminishes, overlapping with a gradual increase in expression of another transporter, potassium-chloride co-transporter 2 (KCC2). KCC2 extrudes chloride from the cell, keeping the basal intracellular chloride at a lower, modest concentration. Thus, when GABA binds to its receptor, chloride enters the cell and hyperpolarizes it, keeping the membrane potential from reaching action potential threshold. Therefore, the relative levels of membrane transporter expression govern chloride concentration and thus the direction of the GABA response. Recent data suggest that other physiological factors also participate in early-life GABA action—in addition to chloride-cation co-transporters, the GABA depolarization-to-hyperpolarization switch is also modulated by the age-related concentration profiles of impermeant anions in the cytoplasm and extracellular matrix [36].

Early transient GABA-mediated depolarization might account, at least in part, for the refractoriness of neonatal seizures to standard ASDs like benzodiazepines (BZDs) and phenobarbital (PHB), both of which increase inhibition by enhancing GABA_A_ receptor-activated chloride channel function in the mature brain. During early development, these agents may not only fail to suppress neonatal seizures but might actually make the seizures worse [37]. The phenomenon of depolarizing GABA action may have wider applicability as well, since in some adult epilepsy models, neurons revert from GABA-induced hyperpolarization to GABA-induced depolarization during epileptogenesis [38] and after head trauma [39]. Moreover, seizure activity may change intracellular chloride concentration and shift the GABA equilibrium potential in the depolarizing direction [40]. 

Lest we conclude that the developing brain is so seizure-prone that it is seizing all the time, it must be remembered that, even during the time window that GABA exerts depolarizing actions, it is also capable of mediating inhibition. The GABA depolarizing effect is not ubiquitous in all brain areas and the transition to a hyperpolarizing action is not sudden; rather, GABA gradually becomes hyperpolarizing as the co-transporter ratio changes and the other mechanisms described above evolve. Furthermore, even in the depolarizing time frame, GABA can mediate some inhibition by a shunting mechanism, whereby a large increase in membrane conductance occurs in response to GABA binding to its postsynaptic receptor. Despite a lack of chloride ion flow in this circumstance, shunting occurs that counters the excitation from activation of nearby α-amino-3-hydroxy-5-methyl-4-isoxazoleproprionic acid (AMPA) and N-methyl-D-aspartate (NMDA)-type receptors [41]. Therefore, dynamic changes in neurotransmitter actions across the lifespan contribute to seizure pathophysiology, and we must be mindful that subtle differences might exist between humans and experimental models.

### 2.3. Antiseizure Drugs May Not Act via the Expected E/I Spectrum

The concept of E/I balance is useful for understanding both pathophysiology and treatment. Indeed, most currently available ASDs work or are designed to address these E/I mechanisms. For example, carbamazepine and phenytoin block sodium channels and reduce repetitive firing, topiramate reduces glutamatergic neurotransmission by blocking AMPA receptors, BZDs and PHB enhance inhibition by increasing chloride (Cl^−^) current flow into cells leading to hyperpolarization, etc. (Table 2). However, not all ASDs work via these classic E/I mechanisms. Levetiracetam (and its analogue, brivaracetam) is a commonly prescribed, broad spectrum ASD with clinical efficacy against both focal and generalized seizures, but does not appear to alter the function of any neurotransmitter receptor or ion channel. Rather, levetiracetam binds to a presynaptic vesicle membrane protein called SV2A, altering the ability of synaptic vesicles to fuse with the presynaptic membrane and release neurotransmitter by exocytosis [42]. Levetiracetam also inhibits presynaptic voltage-gated Ca^2+^ channels and prevents release of Ca^2+^ from intracellular stores, both of which will decrease excitability and reduce transmitter release [43]. While a complete understanding of the mechanisms of levetiracetam action is not yet available, it is clear that this agent acts differently than most ASDs (Figure 2C), paralleling the drug’s unique profile of preclinical seizure suppression—it prevents seizures in chronic epilepsy models induced by kindling or chemoconvulsants such as kainic acid and pilocarpine, but is not effective against acute generalized seizures such as those induced by maximum electroshock or pentylenetetrazole [44,45].

Another example of seizure prevention from an unexpected source is the story of fenfluramine in Dravet syndrome. Clinical series have shown a remarkable efficacy of fenfluramine [46], a serotonin agonist, in this sodium channel disorder in which the primary pathophysiology is thought to be disinhibition by mutations reducing GABA release from interneurons onto excitatory pyramidal cells [19]. The ability of fenfluramine to enhance the activity of only select serotonin receptor subtypes (as well as sigma-1 receptors) opens this new avenue of treatment but still leaves the mechanism by which serotonin reduces excitability unexplained (e.g., possibly by enhancing GABA action) [47].

### 2.4. Metabolic Regulation of Excitability and Epilepsy Requires Expansion of E/I Considerations

Epilepsy is widely viewed as a disorder of neuronal and network excitability. Metabolism, fundamentally important for cell growth, function, and homeostasis, has only recently been appreciated as having a role in neuronal excitability. Not only do seizing brain regions require and utilize excess energy, but genetic or acquired metabolic alterations can also engender seizures, leading to the conclusion that metabolic dysfunction is both a cause and consequence of epilepsy [48]. 

The ketogenic diet (KD) represents a proof-of-principle example of how metabolism affects neuronal excitability and seizure occurrence. This diet, involving a high (4:1) ratio of dietary fat to carbohydrates (by weight), has been used for empiric control of drug-refractory pediatric seizures for almost a century. Efforts to unravel the mechanism(s) by which the KD prevents seizures have been ongoing for more than two decades [49,50]; it is clear that a simple E/I imbalance does not explain the KD’s mechanism of action. Rather, multiple interacting mechanisms are likely in play, involving endogenous anticonvulsants such as adenosine [51], ATP-sensitive K^+^ channels that link metabolism with membrane excitability [52,53], and alteration in biochemical subcellular pathways and energy-producing mechanisms such as mitochondria [48,54,55]. Mutations in genes governing any of the above mechanisms can predispose to seizure generation.

The KD (and its less restrictive form, the modified Atkins diet) mimics the fasting state and produces a state of ketosis by depriving the body/brain of its usual substrate for energy production (glucose), causing a transition to the use of ketones for energy, derived from beta-oxidation of fatty acids. How this metabolic adaptation leads to seizure protection remains unclear. The mechanism can be approached using animal models by considering the effects of high fats or reduced carbohydrates. Early studies posited that ketone bodies per se do not significantly affect GABAergic or glutamatergic neurotransmission, intrinsic membrane properties, or ion channels [56]. It is now appreciated that ketones can affect excitability directly, via modulation of the mitochondrial permeability transition pore [57]. Interestingly, the medium-chain triglyceride (MCT) form of the KD has been shown to have a direct inhibitory effect on AMPA receptors, decreasing fast excitatory neurotransmission [58]. 

The alternative pathway, carbohydrate restriction, can be studied by inhibiting glycolysis with 2-deoxyglucose (2DG). 2DG blocks phosphoglucose isomerase, a key enzyme in glycolysis. In animal models and hippocampal slices, 2DG reduces excitability and suppresses seizures and epileptogenesis [59]. While the mechanism of 2DG action is still being explored, inhibition of glycolysis offers a promising metabolic approach for seizure control. 

Other potential metabolic alterations similarly may ameliorate excitability and seizures and lead to clinical applications. These include calorie restriction, a low glycemic index treatment (LGIT), and an anaplerotic diet, each of which acts at a different target within metabolism (Figure 2D). Calorie restriction mimics the KD by limiting energy availability [60]. The LGIT provides carbohydrates with low glycemic indices, smoothing out the acute elevations in blood glucose levels caused by foods with high glycemic indices [61]. Anaplerosis involves replenishment of tricarboxylic acid cycle components that are depleted when energy demand is high, such as during a seizure [62]. Thus, a wide variety of ways exist by which metabolism can modulate excitability and how metabolism might be manipulated for potential therapeutic benefit.

## 3. Conclusions

This is an exciting era in medicine, with an explosion of new mechanisms (and therefore targets) informing the clinical care of children with epilepsy. While the concept of E/I balance has served well for many decades, the expansion of this paradigm beyond simple excitation and inhibition now opens new avenues for therapy.

## Figures and Tables

**Table 1:** Examples of genes in neurodevelopmental disorders with epilepsy that do not have simple or direct E/I imbalance as a mechanism of action.

GENE	GENE PRODUCT	ROLE	EPILEPSY SYNDROME
*STXBP1*	Syntaxin binding protein 1	Vesicle fusion with presynaptic membrane allowing neurotransmitter release	Ohtahara syndrome
*ARX*	Aristaless-related homeobox protein	Tangential migration of interneurons into the cortical plate	Multiple seizure types, infantile spasms
*CDKL5*	Cyclin-dependent kinase-like 5	Actin cytoskeleton, dendritic arborization, *MeCP2* phosphorylation	Multiple seizure types, infantile spasms
*PCDH19*	Protocadherin 19	Neuron adhesion during migration	Female-restricted epilepsy +/− ID, multiple seizure types, infantile spasms
*UBE3A*	Ubiquitin protein ligase E3A	Targets proteins for intracellular degradation	Angelman syndrome
*PTEN*	Phosphatase and tensin homolog	Tumor and cell growth/migration suppression	Cowden syndrome, focal seizures

*MeCP2*, methyl-CpG-binding protein 2; ID, intellectual disability.

**Table 2:** Mechanisms of selected antiseizure drugs (ASDs).

ASD	Mechanism	E/I Alteration
Phenobarbital	Enhances GABA_A_ receptor function by increasing chloride channel open time	↑ I
Phenytoin	Blocks Na channels	↓ E
Carbamazepine, Oxcarbazepine	Blocks Na channels	↓ E
Valproate	Multiple—enhances GABA action, blocks Na and Ca channels	↓ E, ↑ I
Ethosuximide	Blocks T-type Ca channels	↓ E
Benzodiazepines	Enhance GABA_A_ receptor function by increasing frequency of chloride channel openings	↑ I
Levetiracetam, Brivaracetam	Modulate synaptic vesicle protein SV2A	Unclear
Topiramate	Multiple—enhances GABA action, blocks AMPA receptors and Na channels	↓ E, ↑ I
Vigabatrin	Inhibits GABA transaminase	↑ I
Zonisamide	Multiple—blocks Na and Ca channels, alters neurotransmitter transport	↓ E
Perampanel	Blocks AMPA receptors	↓ E

AMPA, α-amino-3-hydroxy-5-methyl-4-isoxazoleproprionic acid; Ca, calcium; Na, sodium; GABA, gamma-aminobutyric acid; SV, synaptic vesicle; E, excitation; I, inhibition.
